# Rationalizing risk aversion in science: Why incentives to work hard clash with incentives to take risks

**DOI:** 10.1371/journal.pbio.3002750

**Published:** 2024-08-15

**Authors:** Kevin Gross, Carl T. Bergstrom

**Affiliations:** 1 Dept. of Statistics, North Carolina State University, Raleigh, North Carolina, United States of America; 2 Dept. of Biology, University of Washington, Seattle, Washington, United States of America; University of Bristol, UNITED KINGDOM OF GREAT BRITAIN AND NORTHERN IRELAND

## Abstract

Scientific research requires taking risks, as the most cautious approaches are unlikely to lead to the most rapid progress. Yet, much funded scientific research plays it safe and funding agencies bemoan the difficulty of attracting high-risk, high-return research projects. Why don’t the incentives for scientific discovery adequately impel researchers toward such projects? Here, we adapt an economic contracting model to explore how the unobservability of risk and effort discourages risky research. The model considers a hidden-action problem, in which the scientific community must reward discoveries in a way that encourages effort and risk-taking while simultaneously protecting researchers’ livelihoods against the vicissitudes of scientific chance. Its challenge when doing so is that incentives to motivate effort clash with incentives to motivate risk-taking, because a failed project may be evidence of a risky undertaking but could also be the result of simple sloth. As a result, the incentives needed to encourage effort actively discourage risk-taking. Scientists respond by working on safe projects that generate evidence of effort but that don’t move science forward as rapidly as riskier projects would. A social planner who prizes scientific productivity above researchers’ well-being could remedy the problem by rewarding major discoveries richly enough to induce high-risk research, but in doing so would expose scientists to a degree of livelihood risk that ultimately leaves them worse off. Because the scientific community is approximately self-governing and constructs its own reward schedule, the incentives that researchers are willing to impose on themselves are inadequate to motivate the scientific risks that would best expedite scientific progress.

## Abstract 2

Scientific research requires taking risks, so why does much funded scientific research play it safe? This study uses an economic contracting model to argue that this can be explained in part by the non-observability of scientists’ effort and their risk-taking, coupled with the self-organized nature of science.

## Introduction

Scientific inquiry is a risky business. Every experiment, every analysis, every collaboration entails embarking on a path whose destination is uncertain and whose terminus could be a dead end [1]. Not all projects are equally risky, however. In choosing what to work on, scientists have the latitude to embrace risk or to shy away from it. High risk can bring high return; some of the biggest scientific advances arise from risky projects, and credit accrues to investigators accordingly [2,3]. Excessive caution hampers scientific progress, and so scientists, funders of science, and the public all have an interest in encouraging researchers to pursue risky projects. Yet, formal and anecdotal evidence alike suggest that investigators shy away from taking the big risks that may generate the most productive science [4,5]. Even efforts to solicit risky research proposals have met with middling success [5,6]. These observations beg the question: Why don’t the incentive structures in science impel researchers to pursue sufficiently high-risk research?

In this paper, we use a mathematical model to study how scientists’ willingness to pursue risky projects is affected by the non-observability of their actions. By “the non-observability of their actions,” we mean that scientists are not directly rewarded for their effort or for the degree of risk they assume in their research. Instead, investigators are rewarded for the scientific advances that their research yields. However, the uncertain nature of science means that a scientist’s productivity is only stochastically related to the effort they expend and the scientific risk they take on. Thus, in deciding how hard to work and how much scientific risk to embrace, an investigator must weigh the potential for discoveries and acclaim against the chance that their project will fail, leaving them with little to show for their efforts. Here, we aim to understand how the unobservable, or hidden, nature of effort and risk shape investigators’ research strategies and the incentive structures within which they work. Of course, observability is not the only factor that affects investigators’ risk preferences. Franzoni and colleagues [5] have recently reviewed a range of explanations for why science funders and scientists who seek funding may be averse to scientific risk. Elsewhere, we and others have considered how the ex ante nature of grant peer review can deter investigators from proposing projects that their colleagues would consider risky [7–9].

The claim that effort and risk-taking are unobservable to those who ultimately determine how an investigator’s work is rewarded—namely, to the scientific community—is the central premise of our model. While the unobservability of risk-taking and effort is a foundational tenet of the economics of science [10,11], it nevertheless benefits from further justification in the present context. Of course, scientists’ actions, especially their effort, are observable to those around them—their bosses, their colleagues, their family, and their friends. But these are not the people who ultimately determine whether scientists get the university tenure-track job or are honored by a professional society. Instead, career success accrues based on how the scientific community evaluates scientists’ contributions. Thus, it is the scientific community that serves as the ultimate arbiter of investigators’ professional success, and this community does not have the means (because of its physical dispersion) or perhaps even the interest to monitor investigators’ effort. Indeed, to a first approximation, scientists’ careers rise and fall based on their CVs, and CVs list only scholarly outputs, not hours worked or risky undertakings gone bust. (At first glance, it may seem that scientists’ supervisors determine professional rewards and could monitor effort if they were so inclined. Yet, in academic science, supervisors primarily reward researchers based on the esteem in which those researchers’ contributions are held by scientific community. Thus, supervisors act in response to the community’s judgment, not separately from it.) This set of circumstances, and thus our model, pertain most directly to investigators who work individually or in small teams on projects that may take a few months or a few years, or at most a single career. They do not describe, and thus our model does not capture, large infrastructure projects such as space exploration or high-energy physics where the need to coordinate huge teams makes effort plainly visible to the scientific community in which those teams operate.

Formally, we study investigators’ risk-taking behavior using the economic framework of a hidden-action problem. Hidden-action, or moral hazard, problems are most often used to study the tensions that arise when a “principal” writes a contract to hire an “agent” to work on the principal’s behalf [12,13]. In our setup, individual investigators are the agents while the scientific community collectively plays the role of the principal. We can treat the scientific community as the principal because academic science is a self-organized pursuit, in which scientists operate within institutions—hiring norms, systems for funding, tenure standards, etc.—that they have designed for themselves and that endure only with their continued blessing. While this is undoubtedly a simplification, it hews close enough to reality to expose fundamental tensions at work in motivating scientists to do risky research. (When necessary to avoid ambiguity, we refer to scientists acting collectively as the “scientific community” and individual scientists acting in their own best interest as “scientists,” “investigators,” or “researchers.”) The “contract” that the scientific community establishes is not an actual contract, of course, but instead represents the traditions that the community establishes for rewarding investigators for their discoveries. In other words, we study how much prestige the scientific community assigns to a publication in (say) *PLOS Biology* as opposed to a publication in a competing journal, or to a failed research attempt that generates no publication at all.

In establishing these traditions, the scientific community must collectively solve the following problem. The community receives resources—jobs, funding, prestige, etc.—from the public to support the scientific endeavor in exchange for the knowledge that the community produces [14]. The community seeks to distribute these resources among its members in whatever manner its members favor most, but in doing so, it is constrained in the following ways. First, because only scientists’ discoveries (e.g., publications) are observable, the community’s tradition for distributing rewards can only be based on the scientific advances that researchers produce. Second, the community is not free to bestow unlimited rewards on its members. Instead, the total volume of rewards is linked to the public’s aggregate support of science. Third, while individual researchers are not disposed to embrace or to shy away from scientific risk for its own sake, researchers are averse to putting their livelihoods at risk in the usual way that economic actors are assumed to be averse to livelihood risk.

To sum, we model how a scientific community establishes a tradition for rewarding discoveries that motivates investigators to work hard and to take scientific risks while also protecting researchers’ livelihoods from the vicissitudes of scientific chance. For the sake of comparison, we also solve the same contracting problem from the perspective of a (fictitious) social planner who cares most about optimizing scientific progress and who only cares secondarily about investigators’ preferences. Our goal is to understand how the hidden nature of researchers’ actions affects the scientific community’s ability to motivate researchers to take scientific risks, if it does at so all, and to understand any consequences for the aggregate productivity of the scientific community.

The most closely related modeling work to this analysis seems to be Manso’s study of the tension between risk-taking and effort in the context of executive compensation [15]. The scientific community’s problem also resembles an optimal taxation problem, in the sense that the community redistributes the fruits of scientific progress in a way that maximizes researchers’ individual well-being while preserving the incentive for investigators to make important discoveries [16,17]. Franzoni and colleagues [5] provide a recent qualitative analysis of the reasons why investigators often eschew risky research.

## Mathematical model

Our mathematical framework builds from the economic theory of hidden-action models [12,13]. While a familiarity with hidden-action models is not necessary to understand the setup, Box 1 provides a very brief introduction to hidden-action models for curious readers. Ch. 4 of [16] provides a more thorough introduction.

**Box 1. Hidden-action models**

Hidden-action, or moral-hazard, models are tools for analyzing tensions that arise when one party—typically dubbed the “principal”—hires a second party—the “agent”—to do costly work on the principal’s behalf. To do so, the principal seeks to propose a compensation scheme (the contract) that the agent will find agreeable and that will motivate the agent to put forth work. The principal’s ability to write this contract efficiently is hindered by two key tensions. The first tension is an information asymmetry: The effort that the agent invests in their work is known only to the agent, and, consequently, the principal cannot compensate the agent directly for their effort. Instead, the agent’s effort yields an output. The output is publicly observable and can be contracted upon, but the agent’s effort only stochastically determines their output. (Typically, greater effort yields a stochastically larger output.) The second tension arises because a stochastically determined output creates risk, and the principal and the agent are presumed to have different risk tolerances. Usually, the principal is thought to be more willing to bear risk than the agent because the principal might enter into multiple contracts with several agents at once, while the agent only enters into a single contract. Alternatively, the principal might have a limited ability to penalize the agent for a low output, which creates a similar tension.

Principal-agent models also assume that the principal has the bargaining power. In other words, the principal is free to propose a contract that specifies the agent’s compensation (their “wages”) as a function of the agent’s output. The agent then chooses whether or not to accept the contract, and if they accept it, the agent also decides privately how much effort to expend.

The principal’s problem is then to find the contract that yields the best outcome for the principal, under the constraints that the contract cannot pay the agent directly for their effort and that the agent is not obligated to accept the contract. The classical result is that the principal’s preferred contract leaves the principal worse off and elicits less effort from the agent compared to the contract that the principal could propose if the agent’s effort were directly observable or if the principal and agent were equally tolerant of risk.

Hidden-action settings are routine in everyday life. Classic examples include insurance contracts, managerial contracts, and contracts for specialized labor. The setting that we analyze in this paper differs from the standard hidden-action setting in two key ways. First, the principal in our case is the scientific community, and, thus, the principal’s interests are aligned with the agents’ (researchers’) interests. Second, the researchers’ action has two distinct components: how hard they work (their effort) and the degree of scientific risk they take on. Both components of the action affect the researcher’s output, but only effort is costly to the researcher. Our setting aligns with the classic hidden-action setup because the scientific community (by virtue of its size) can tolerate more risk than individual researchers, and because the community seeks a contract, or tradition, for rewarding the researchers for an output (scientific productivity) that is stochastically determined by the researchers’ actions.

Our model envisions a scientific workforce that consists of a unit mass of investigators. To get off the ground, we make two substantial assumptions. First, we assume that all scientists are alike. Second, we consider a one-shot setting in which investigators attempt a single study instead of building a portfolio of complementary studies. Clearly, both of these assumptions are unrealistic. Their purpose here is to allow us to focus on the key strategic dilemma that the community faces when attempting to motivate both risk-taking and hard work. Relaxing either or both of these assumptions complicates the community’s task in interesting ways that would be ripe for subsequent analysis. We examine both assumptions further in the Discussion.

When an investigator pursues a study, they must decide both how risky of a project to pursue and how much effort to invest. Let *r* represent a study’s scientific risk and let *e* represent an investigator’s effort. We will let both *r* and *e* take values in [0,1], with larger values of *r* and *e* corresponding to greater risk and more effort, respectively. Together, the risk–effort pair (*r*, *e*) forms an investigator’s action. We assume that there is no direct cost to scientific risk, in the sense that risky projects are no more or less onerous than safer ones. (In reality, it may be that riskier projects are also more onerous, which, if true, would exacerbate the tension studied here.) By contrast, effort is costly to investigators, such that an investigator who expends effort *e* incurs a disutility cost *c(e)*, with *c*(0) = 0, *c*′>0, and *c*′′>0. The convexity of *c* can be motivated by assuming that researchers obtain decreasing marginal returns to leisure, a standard assumption in labor economics [18].

Each study generates an outcome that has a scientific value *v*≥0. The distribution of *v* depends on the investigator’s action. Some studies yield unpublishable outcomes, in which case they contribute nothing to science and have value *v* = 0. Publishable outcomes have strictly positive value (*v*>0), with larger values of *v* corresponding to more valuable outcomes. We assume that the values of published outcomes are continuously distributed on *v*>0. Thus, for any action, the corresponding distribution of *v* includes a probability mass at *v* = 0 and a probability density on *v*>0. Because the distribution of *v* has both discrete and continuous components, it is most naturally represented by the cumulative distribution function (cdf) *F*(*v*) = Pr{*V*≤*v*}, where *F*(0) is the probability of an unpublishable outcome. Let *F*(*v*;*r*,*e*) denote the cdf of *v* corresponding to the action (*r*,*e*).

Because we describe the distribution of *v* by its cdf, we use ∫ *g*(*v*) *F*(*dv*;*r*,*e*) to denote the expectation of a function *g*(*v*) when the distribution of *v* is given by *F*(*v*;*r*,*e*). Accordingly, let *s*(*r*,*e*) = ∫ *v F*(*dv*;*r*,*e*) denote the expected scientific productivity of an investigator who takes action (*r*,*e*). Because we have assumed that all investigators are alike, *s*(*r*,*e*) also gives the aggregate per capita productivity of the scientific community when every investigator takes action (*r*,*e*). We assume that the scientific community is large enough that the stochasticity in the community’s aggregate productivity is negligible. Let $\left(\hat{r},\hat{e}\right)$ be the action that maximizes scientific productivity, and let $\hat{r}(e)$ be the level of risk-taking that maximizes scientific productivity for a given level of effort *e*. We assume that $\left(\hat{r},\hat{e}\right)$ is unique and that $\hat{r}(e)$ is unique for any value of *e*.

**Fig 1:**
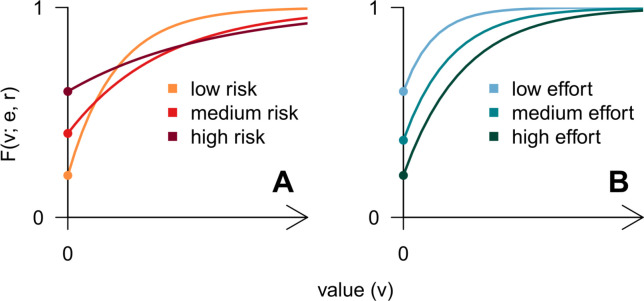
Risk and effort have differing effects on the distribution of a project’s value. Each panel shows the cumulative distribution function *F*(*v*;*r*,*e*) of a project’s value *v*. Values at *v* = 0 correspond to the probability of generating an unpublishable outcome. (**A**) Increasing scientific risk increases the probability of getting an unpublishable outcome but also increases the expected value of publishable outcomes. (**B**) Increasing effort decreases the probability of getting an unpublishable outcome and increases the probability of publishing an outcome with a large value.

We now specify how the distribution of *v* depends on the investigator’s action. Consider risk first. While there are many facets of scientific risk [1,19,20], here, we assume that scientific risk has the following properties. First, an increase in risk increases the probability that an investigator will unluckily generate an unpublishable outcome [21] and will have nothing to show for their efforts (Fig 1A). Because scientific risk has no natural units, we might as well equate a project’s scientific risk with the probability that a study generates an unpublishable outcome when an investigator gives full effort, that is, *F*(0; *r*, e = 1) = *r*. Second, an increase in risk increases the conditional expectation of an outcome’s scientific value given that it is publishable. Third, we assume that *s*(*r*,*e*) is strictly concave in *r* for any value of *e*.

Now consider effort. Zero effort (*e* = 0) guarantees zero science (*v* = 0), that is, *F*(0; *r, e* = 0) = 1. Second, for any scientific risk and any scientific value *v*, increasing effort strictly increases the the chance of obtaining an outcome at least as valuable as *v* (Fig 1B). In other words, increasing effort shifts the graph of *F(v)* downwards and to the right. (Put differently, increasing effort increases *v* in the sense of first-order stochastic dominance.) This implies that *s*(*r*,*e*) increases in effort for any value of *r*.

Finally, we require a few technical regularity conditions on *F*(*v*;*r*,*e*) that are described more fully in S1 Appendix. The most substantive of these is a monotone likelihood ratio property (MLRP) with respect to both risk *r* and effort *e*. The MLRP, with respect to risk, states that among publishable outcomes, a higher-valued publication provides more evidence of greater risk than a lower-valued publication provides. With respect to effort, the MLRP states that among all studies (publishable or not), a larger value of *v* provides at least as much evidence of greater effort than a smaller value of *v* provides.

We now turn to the problem that the scientific community faces in establishing its tradition for rewarding investigators for their discoveries. Society provides resources to the scientific community in return for scientists’ collective progress. These resources include all forms of compensation, such as income, jobs, prestige, etc. We assume that the total resources are directly linked to the aggregate scientific productivity [14] and write the per capita resources (the community’s resource “budget”) as *B(s)*, with *B*(0) = 0 and *B*′>0. The resource budget represents a longstanding arrangement between the public and the scientific community that emerges over many rounds of the one-shot process that we consider.

The scientific community distributes these resources among its members based on the discoveries that researchers generate. Let *w*(*v*)≥0 denote the resource reward (or “wages”) that a scientist receives in return for a scientific outcome of value *v*. We call *w*(∙) the community’s “contract,” and we assume that *w*(∙) is differentiable for *v*>0. A scientist’s utility increases by *u*(*w*)≥0 if they receive wage *w*. We assume that *u*(0) = 0, *u*′>0, and *u*′′<0, so that scientists are strictly risk averse with respect to their wages. A scientist who chooses action (*r*,*e*) while facing contract *w*(∙) obtains the payoff

π(r,e,w(·))=∫u(w(v))F(dv;r,e)−c(e). (1)

A contract *w*(∙) is said to implement the action (*r*,*e*) if an investigator facing *w*(∙) maximizes their payoff by choosing that action.

Two comments are in order before proceeding. First, it is useful to recall that the researchers’ “payoff” is merely a mathematically convenient device for capturing preferences [22]. In other words, to say that one action has a higher payoff than another is simply a way of stating that the action with the higher payoff is preferred to the action with a lower payoff. Second, by associating the researchers’ payoff with the professional returns that the scientific outcome brings, we follow the argument that scientists, in addition to following their own curiosities, want to do work that is rewarded [23].

The scientific community’s problem is to find a contract *w*(∙) that maximizes its members’ payoff while implementing an action that generates enough aggregate scientific productivity to justify the public’s investment. In other words, the community’s problem is

$$\underset{r,e,w(\cdot )}{max}\pi (r,e,w(\cdot ))$$ (2)

subject to the following constraints. First, we assume that the contract must reward high-value science at least as handsomely as it rewards lower-value science. That is, the contract must satisfy the monotonicity constraint

$$\begin{array}{l} \;w(0)\leq \underset{v↓0}{lim}\;w(v)\\ w^{\prime }(v)\geq 0\;for\;v>0. \end{array}$$ (MC)

Second, because the total rewards cannot exceed the resource budget, the contract and the action it implements must satisfy the budget constraint

∫w(v)F(dv;r,e)≤B(s(r,e)). (BC)

Third, the contract must implement the action. That is, the contract must satisfy the incentive constraint

$$(r,e)\in \underset{r\prime ,e\prime }{\mathrm{argmax}}\;\pi (r\prime ,e\prime ,w(\cdot )).$$ (IC)

Both the effort and risk components of the IC optimize over a continuum of possible actions and thus entail an infinite number of constraints. We follow the usual procedure of analyzing instead the relaxed problem in which both components of the IC are replaced by the corresponding first-order conditions

$$\frac{\partial }{\partial r}\pi (r,e,w(\cdot ))=0$$ (RC)

and

$$\frac{\partial }{\partial e}\pi (r,e,w(\cdot ))=0.$$ (EC)

We call these the risk constraint (RC) and the effort constraint (EC), respectively. While cautions apply to using a first-order condition [24–26], we assume that the second-order conditions needed to justify the use of the RC and EC hold for any *w*(∙) that satisfies the MC. Write the action implemented by the community’s optimal contract as $\left(\tilde{r},\tilde{e}\right)$.

Hidden-action problems also typically involve a participation constraint. Suppose that an investigator may leave science to pursue an outside option that provides the reservation payoff *π*_*R*_≥0. The participation constraint (PC) would then read *π*(*r*,*e*,*w*(∙))≥*π*_*R*_, assuming that investigators participate at indifference. To ensure that there are at least some contracts that make it worthwhile for investigators to participate in science, we assume that there is at least one action for which an investigator’s payoff under the “soft-money” contract *w*(*v*) = *vB*(*s*)/*s* satisfies the PC. Under this assumption, the PC does not factor into the scientific community’s problem.

We consider two benchmark settings for comparison. The first is the hypothetical full-information scenario in which the investigators’ actions are observable. In this scenario, the community can mandate that investigators must take a particular action to receive the rewards offered by the contract. The full-information solution solves (2) subject to only the MC and BC. The second benchmark is the problem that would be faced by a hypothetical social planner whose primary objective is to maximize scientific productivity. This planner’s problem is

$$\underset{r,e,w(\cdot )}{max}s(r,e)$$ (3)

subject to the MC, BC, RC, and EC. (The PC may also constrain the social planner. When it does, the social planner finds the contract that maximizes scientific productivity while just providing the investigators with a payoff of *π*_*R*_. Because the social planner’s problem is only a benchmark and not the primary focus of this article, we do not explore the consequences of the PC for the social planner any further.) We will see that the social planner may have several contracts available that implement the productivity-maximizing action $\left(\hat{r},\hat{e}\right)$. In these cases, we assume that the social planner seeks the contract that maximizes the investigators’ payoff while implementing $\left(\hat{r},\hat{e}\right)$.

## Analysis

### Full-information benchmark

To begin, consider the full-information benchmark in which investigators’ actions are observable. In this case, the community is best off paying everyone who takes the contracted action the same wage regardless of the scientific output that their research yields. The intuition here is clear: If actions are observable, then the community bears the scientific risk and insures investigators fully, because an investigator who takes the mandated action but produces a weak or unpublishable result has manifestly just been unlucky.

With full insurance, the payoff-maximizing effort (and, thus, the effort that the community demands of its members) will depend on the details of *B*(∙), *s*(∙,∙), and *c*(∙) and will not necessarily maximize scientific productivity. However, regardless of the optimal effort level, the community will mandate the level of scientific risk-taking that maximizes scientific productivity for the optimal level of effort. In other words, under full information, scientific risk-taking maximizes scientific productivity for the level of effort that is best for the community.

The same logic prevails in an intermediate scenario in which only risk-taking is hidden but effort is observable. In this scenario, the community can still fully insure the investigators, and, thus, the community settles on the same contract as the full-information benchmark. Consequently, scientific risk-taking is not distorted when effort is observable, regardless of whether scientific risk is observable.

### Second-best outcomes when researchers’ actions are hidden

We now return to the setting when both scientific risk-taking and effort are hidden. Ideally, we would like to identify the precise conditions under which scientific risk-taking is or is not distorted away from its productivity-maximizing level. Unfortunately, such a result eludes us. In the absence of a sharp result, we proceed in two complementary directions. First, we show that scientific risk-taking is distorted downwards in the special case when effort scales the probability that an outcome is publishable. Second, we present a numerical example that suggests that risk-taking will still be distorted downwards in the more complex and realistic case in which effort affects both the probability that an outcome is publishable and the distribution of *v* for publishable outcomes. We conclude by arguing why we expect the intuition in the special case to carry over to the richer case.

#### Special case: Effort scales the probability of publication

Here, we impose the additional restriction that effort only affects the probability of publication. More specifically, we assume that the probability of generating a publishable outcome scales linearly with *e* and thus equals *e(*1*-r)*. Let *F*(*v*;*r*) give the cdf of *v* under full effort. Then, the cdf of *v* under any effort in this case is then given by

F(v;r,e)=1−e+eF(v;r). (4)

In this special case, the community’s tradition always distorts risk-taking downward from its productivity-maximizing level, that is, $\tilde{r}<\hat{r}(\tilde{e})$ as long as *F*(*v*;*r*) meets a few additional mild technical conditions. A mathematical proof appears in S1 Appendix.

To sketch the intuition behind the result, we appeal to a standard result from moral-hazard theory, which is that the optimal contract for implementing a particular action rewards outcomes in proportion to the evidence or “good news” that those outcomes provide for that action [12,13]. When effort is hidden, researchers cannot be fully insured against the risk of a nonpublishable outcome; if they were, they would have no incentive to work hard. To create an incentive for hard work, the community’s tradition must, at a minimum, furnish more handsome rewards to researchers who publish than to researchers who don’t. This reward premium for publication forces researchers to bear some livelihood risk, decreasing their payoff.

While a reward premium for publication is necessary to motivate effort, it simultaneously discourages scientific risk-taking by penalizing researchers who generate unpublishable results. The community must counteract the risk-discouraging effects of a publication premium by rewarding higher-value publications more handsomely, thus restoring an incentive to pursue risky projects. Yet, the uneven reward tradition needed to motivate risk-taking creates its own drag on researchers’ payoff by exposing them to additional livelihood risk. Because of this drag, the community settles for a more egalitarian tradition that balances partial protection from livelihood risk against the reduced productivity of the more conservative science that this tradition implements.

Incentives for risk-taking and effort will always be in tension at *v* = 0, because an unpublishable result provides “good news” about an investigator’s risk-taking but “bad news” about their effort. In the special case of Eq 4, this tension at *v* = 0 is the entire interaction between motivating effort and motivating risk-taking, and, thus, a tradition that encourages effort necessarily discourages risk-taking. This guarantees that under Eq 4, the community’s preferred tradition must distort risk-taking downwards from its productivity-maximizing optimum.

To illustrate with a numerical example, suppose that publishable outcomes have an exponentially distributed scientific value with mean *r*. Thus, scientific productivity *s*(*r*,*e*) = *er*(1−*r*), and the productivity-maximizing action is $\left(\hat{r}=1/2,\hat{e}=1\right)$. Indeed, the productivity-maximizing scientific risk for any effort is $\hat{r}(e)=1/2$. Suppose that an investigator’s disutility cost of effort is given by *c*(*e*) = 0.1*e*^2^ and their utility derived from wages is given by $u(w)=\sqrt{w}$. Finally, assume that the community’s resource budget is linear in the aggregate scientific productivity, *B*(*s*) = *s*.

**Fig 2:**
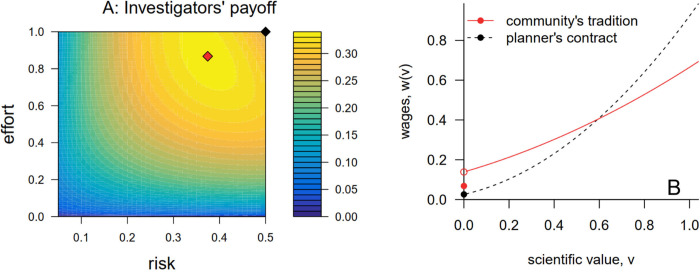
The scientific community’s tradition decreases both risk-taking and effort away from their productivity-maximizing actions while rewarding scientific contributions more evenly. This figure shows the solution to the numerical example described in the text when *F*(*v*; *r*, *e*) is given by Eq 4. (**A**) The investigators’ payoff at the optimal tradition, or contract, for implementing each possible action. The red diamond shows the action that yields the largest possible payoff and, hence, the community’s preferred tradition. The black diamond shows the payoff that the investigators would receive from a social planner who seeks first to maximize scientific productivity and secondly to optimize investigators’ welfare. (**B**) The reward tradition established by the community (red curve) and the contract that a social planner would favor (black). The community’s preferred tradition distributes rewards more evenly among investigators than the the social planner’s contract does. The code to generate this figure can be found in https://zenodo.org/records/12532039.

The community’s preferred tradition and the social planner’s benchmark for this numerical example are illustrated in Fig 2. Note that the community’s problem (Eq 2) can be rewritten as

$$\underset{r,e}{max}\underset{w(\cdot )}{max}\pi (r,e,w(\cdot )),$$ (5)

thus decomposing the community’s problem into 2 subproblems: an “inner” maximization that finds the best contract for each action, and an “outer” maximization that finds the action with the best payoff. Fig 2A shows the investigators’ payoff at the solution to the inner maximization of Eq 5 for each action; that is, it shows the highest payoff that the investigators can receive for each action. The solution to the outer maximization in Eq 5 is shown by the red diamond in Fig 2A. This action and the corresponding reward tradition (Fig 2B) solve the community’s problem.

In this example, a social planner can institute a contract that impels researchers to take the productivity-maximizing action. The social planner does this by solving the inner maximization of Eq 5 for $\left(\hat{r},\hat{e}\right)$, subject to the same constraints that the community faces. The community’s reward tradition distributes rewards more evenly among its members than the social planner’s contract (Fig 2B). The community’s tradition reduces scientific productivity relative to the social planner’s contract ($s(\tilde{r},\tilde{e})\approx 0.203$ for the community’s tradition versus $s(\hat{r},\hat{e})=0.25$ for the social planner’s contract), but it leaves the investigators better off.

Although it is not shown in Fig 2, the full-information benchmark in this example would mandate that each investigator take the productivity-maximizing action and pay each investigator who does so a wage equal to the per capita scientific productivity (*w* = 0.25). This would give the investigators a greater payoff (*π* = 0.4) than they would receive under any hidden-action scenario.

#### Richer case: Effort affects both the probability of publication and the distribution of *v*

**Fig 3:**
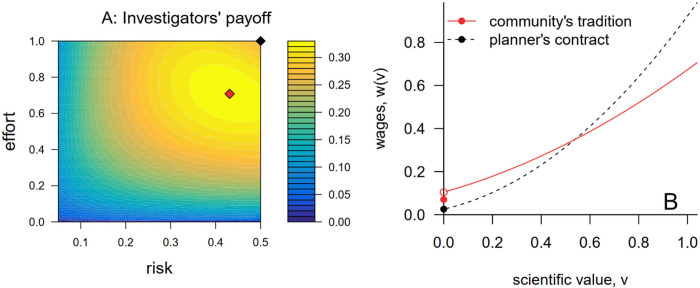
The scientific community’s tradition decreases risk-taking and effort away from their productivity-maximizing actions in a richer model in which effort also affects the probability distribution of publishable results. This figure is structured identically to Fig 2 but pertains to the case when effort affects both the probability of generating a publishable outcome and the distribution of *v* for publishable outcomes. See the text for model details. The code to generate this figure can be found in https://zenodo.org/records/12532039.

The additional structure of Eq 4 is sufficient to guarantee that scientific risk-taking is distorted downward from its productivity-maximizing level. However, Eq 4 is not necessary for risk-taking to be distorted downward, and, indeed, we expect that the same intuition is likely to prevail in the more complex and realistic case when effort can also affect the distribution of *v* for publishable outcomes. To illustrate, consider a variation of the previous numerical example. Now, suppose that the probability of generating a publishable outcome is given by $\sqrt{e}(1-r)$ and outcomes that are publishable have an exponentially distributed scientific value with mean $r\sqrt{e}$. Thus, as before, scientific productivity *s*(*r*,*e*) equals *er*(1−*r*), and the productivity-maximizing action remains $\left(\hat{r}=1/2,\hat{e}=1\right)$. As before, we continue to assume $c(e)=0.1e^{2},\;u(w)=\sqrt{w}$, and *B*(*s*) = *s*. Fig 3 shows that in this more complex case the community’s optimal contract continues to distort both scientific risk-taking and effort downwards from their productivity-maximizing values.

The results in Fig 3 suggest that the community will continue to adopt a reward tradition that encourages researchers to play it safe even when effort has more complex effects on the distribution of outcomes that investigators’ research generates. We expect that this is a broadly general result that will apply for most reasonable choices of *F*(*v*;*r*,*e*). The intuition here is the same as before: Motivating researchers to expend costly effort requires creating a reward premium for publishable outcomes, but this premium explicitly discourages risk-taking. We can’t rule out the possibility that risk-taking will not be distorted in the more general model, because it is conceivable that “good news” about effort and risk-taking might be strongly enough aligned for publishable outcomes (*v*>0) to counteract the tension at *v* = 0, thereby making it less costly to motivate effort when investigators pursue riskier-than-optimal science. Although this scenario seems far-fetched to us in practice, nothing in the mathematical structure of the model seems to rule out the possibility. (Note that our lack of a sharp result in the absence of Eq 4 is only a statement of our own inability to obtain such a result. We haven’t yet found a counter-example under which risk-taking is not distorted downwards, so we may have just failed to find an appropriate proof.)

## Discussion

On the face of it, scientists as a group seem to face dilemma that they are unable to solve. On the one hand, risky research generates the groundbreaking advances that expand our knowledge most rapidly. On the other hand, scientists seem either unable or unwilling to devise institutions that motivate investigators to embrace the scientific risks that would lead to the most rapid progress [5]. Our analysis here suggests that this state of affairs can be explained at least in part by the interaction between two key structural elements in science: the unobservability of risk and effort on the one hand, and the self-organized nature of science on the other. If either of these elements were reversed—if either risk and effort could be verifiably documented, or if science was governed by a hypothetical social planner with the authority to allocate prestige unilaterally—scientists could either motivate themselves or be motivated by the social planner to implement the productivity-maximizing scientific risk. The mechanisms would be quite different, however; in the former case, scientists would be rewarded directly for their risk and effort choices, while in the latter case, the social planner would heap prestige on investigators who generate the most groundbreaking discoveries while reducing the prestige awarded for more incremental advances.

But neither option is available. We have argued earlier about the hidden nature of risk and effort, ruling out the possibility of awarding prestige based on either. We have said less about why a social planner does not emerge to organize (academic) science. For one, a social planner cannot emerge because science is a specialized pursuit, and assessing the scientific value of discoveries requires a close knowledge of the field. Thus, a hypothetical social planner would be hamstrung by being forced to rely on the guidance of the investigators to determine how to value outcomes, and as such would be no more than a conduit for the investigators’ collective judgements [27]. In other words, they wouldn’t be a planner at all.

Why doesn’t the scientific community simply adopt the scheme that a social planner would advocate? They don’t because the social planner’s scheme leaves the scientists worse off, despite optimizing scientific progress. The social planner’s scheme demoralizes the investigators because it places the investigators at too great a risk of having little to show for their efforts if their scientific risk does not pay off. The scientific community can do better by weakening, though not eliminating, the disparity in prestige awarded for groundbreaking versus incremental outcomes, thus preserving the incentive for investigators to take on some scientific risk while also protecting their livelihoods if the scientific risk doesn’t pan out.

The tension we study here helps to explain some features of the scientific ecosystem, which might otherwise seem perverse. Consider scientific funding. Many funding bodies have programs dedicated to funding risky research. Yet, those funders also ask investigators to report the research outputs generated by prior funding awards. While few would question that a researcher’s past productivity provides useful information to reviewers evaluating new proposals, it is still worth nothing that funders discourage risk-taking when they make prior productivity a de facto requirement for subsequent funding. On its face, this practice seems to send a mixed message to investigators. Yet, such a mixed message may be a reasonable compromise for a community that can only verify a researcher’s outputs. In other words, while the scientific community may be perpetually frustrated by its inability to impel investigators to take bigger risks, this frustration is not necessarily evidence of poor institutional design; it may instead be an unavoidable consequence of information asymmetries inherent in the scientific endeavor.

In some corners of science, recent efforts to reform the publication process such as Registered Reports [28,29] and venues for publishing null results may decouple scientific risk from livelihood risk, thus making high-risk research more attractive to investigators. It will be exciting to see how these nontraditional publication pathways affect scientific activity as researchers build experience with them. Registered Reports, in which researchers can obtain in-principle acceptance of publication pending faithful execution of a peer-reviewed study design, provide an especially interesting case. While Registered Reports may protect researchers against the risk of obtaining a null result, the need to obtain peers’ blessing at the design stage may simultaneously disfavor studies that are risky in another sense, that sense being an investigators’ willingness to leverage their private scientific beliefs when those beliefs depart from the mainstream [9]. This dynamic hints at the likely complex interactions between different facets of scientific risk. It is also worth noting that these nontraditional publication pathways may de-risk some forms of scientific activity, such as surveys, clinical trials, and replication studies, more effectively than other types of work, such as mathematical or theoretical studies.

Our analysis makes a number of simplifications, each of which provide an opportunity for further research. Perhaps most substantively, we have assumed that all investigators are alike. In science, researchers differ in many ways that affect how they design their research programs, including their abilities and their predisposition to take scientific risks [30]. These differences are only privately known to the individual investigators, at least initially. In contracting theory, private differences among agents further complicate the principal’s task through the phenomenon of “adverse selection,” in which the principal’s inability to know agents’ type results in additional distortions away from efficient outcomes. Moreover, the effects of adverse selection compound when investigators also make hidden-action decisions [16]. Without further study, we can only speculate about how these interactions may play out in science, although it seems likely that adverse selection complicates the task of designing a reward scheme that motivates researchers to pursue the type of research at which they excel.

Second, our assumption that researchers are alike makes it easy to determine the scientific community’s objective, because the contract that is best for one researcher is also best for all. In reality, different types of researchers will prefer different reward schemes. Understanding how the community resolves these differences requires an additional understanding of the internal politics of science that eludes the authors.

Our model also simplifies the community’s task by considering only a one-off setting. In reality, of course, researchers build their careers and their reputation through a series of projects, thus allowing investigators to build a research portfolio that may include both risky endeavors and safer bets. Further, the act of doing science is not as simple as our model envisions. In reality, projects evolve, and investigators routinely make operational decisions throughout a project’s lifetime that steer it toward its conclusion. Perhaps one of the most helpful skills in science is the ability to make the proverbial lemonade from lemons, that is, to generate useful science from a risky effort gone bust. Finally, in the long run, a researcher’s output begins to yield information about her abilities relative to those of other investigators, introducing an interaction between one’s career stage and the adverse selection mentioned above that only complicates matters further [31]. All these realities intrude on the simple setting we have analyzed here in ways that will require future work to unravel.

These caveats notwithstanding, the dynamic that we have explored here is inescapable. In deciding how to reward discoveries, the scientific community must contend with the fact that reward schemes that motivate effort inherently discourage scientific risk-taking and vice versa. Because the community must motivate both effort and scientific risk-taking, and because effort is costly to investigators, the community inevitably establishes a tradition that encourages more conservative science than would be optimal for maximizing scientific progress, even when risky research is no more onerous than safer lines of inquiry.

## Supporting information

S1 Appendix Mathematical details. (PDF)

## Abbreviations

BC: budget constraint
cdf: cumulative distribution function
EC: effort constraint
IC: incentive constraint
MLRP: monotone likelihood ratio property
MC: monotonicity constraint
PC: participation constraint
RC: risk constraint
