# Supplementary material for: Rationalizing risk aversion in science: Why incentives to work hard clash with incentives to take risks
Source: PLoS Biol. 2024 Aug 15;22(8):e3002750. doi: 10.1371/journal.pbio.3002750 (PMC11326573; doi:10.1371/journal.pbio.3002750)
Supplement: S1 Appendix — (PDF) [file pbio.3002750.s001.pdf]

S1 APPENDIX: MATHEMATICAL DETAILS

This appendix contains a proof of our main result, details about the monotone likelihood ratio property, and numerical examples. We begin by analyzing the full-information solution to the community's problem, and then proceed to analyze both the general model and the special case of eq. 4. Details for the numerical examples follow.

Throughout, we take the standard step [16] of re-writing the scientific community's problem in terms of the wage-utility schedule $\bar{u}(v) = u(w(v))$. Let $h(\cdot) = u^{-1}$ give the wages needed to achieve utility u, with $h(0) = 0$, $h' > 0$, and $h'' > 0$. To simplify notation, we write the scientists' payoff π as a function of their action (r, e) and the wage utility schedule $\bar{u}(v)$. We also use $f(v; r, e)$ to denote the density of $F(v; r, e)$ with respect to a measure that sums counting measure at $v = 0$ and Lebesgue measure on $\mathcal{R}$. Use subscripts to denote partial derivatives, e.g., $f_r(v; r, e) = \partial f(v; r, e)/\partial r$. Unless otherwise noted, all integrals are with respect to $v \geq 0$. Write the community's (per capita) resource budget under action (r, e) as $\bar{B}(r, e) = B(s(r, e))$.

We take the usual step of separating the problem into two sub-problems [13], as shown in eq. 5. The first sub-problem, or inner maximization in eq. 5, solves for the contract that maximizes the community's payoff when implementing any action (r, e), subject to the same constraints as the full problem. Write the contract that solves this sub-problem as $\bar{u}^{(r,e)}$, that is,

$$\bar{u}^{(r,e)}(\cdot) = \arg\max_{\bar{u}(\cdot)} \pi(r, e, \bar{u}(\cdot)). \tag{S1.1}$$

This sub-problem has a linear objective and a convex constraint space, so any solution that satisfies the Kuhn-Tucker conditions will be a global optimum [32, Thm. M.K.3]. The second sub-problem, or outer maximization in eq. 5, finds the action that maximizes the payoff $\pi(r, e, \bar{u}^{(r,e)}(\cdot))$, that is, it solves

$$\max_{r,e} \pi(r, e, \bar{u}^{(r,e)}(\cdot)). \tag{S1.2}$$

S1.1. Monotone likelihood ratio property

The monotone likelihood ratio property (MLRP) is a regularity condition on $F(v; r, e)$. For risk, the MLRP states that a higher-valued publication provides more evidence of greater risk than a lower-valued publication provides. In notation, this writes as

$$\frac{\partial}{\partial v}\left[\frac{f_r(v; r, e)}{f(v; r, e)}\right] > 0 \tag{S1.3}$$

for $v > 0$. An equivalent statement of the MLRP for r is that for any two values $v_2 > v_1 > 0$, any two risk levels $r_2 > r_1$, and any e, then

$$\frac{f(v_1; r_2, e)}{f(v_1; r_1, e)} < \frac{f(v_2; r_2, e)}{f(v_2; r_1; e)};$$

this expression accords more closely with the name "monotone likelihood ratio". Note that the MLRP for risk does not extend to $v = 0$. Indeed, because $F_r(0; r, e) + \int_{v>0} f_r(v; r, e)\, dv = 0$, $F_r(0; r, e) > 0$ and eq. S1.3 together imply $\lim_{v\downarrow 0} f_r(v; r, e) < 0$. Thus

$$\lim_{v\downarrow 0} \frac{f_r(v; r, e)}{f(v; r, e)} < 0 < \frac{F_r(0; r, e)}{F(0; r, e)}. \tag{S1.4}$$

With respect to effort, the MLRP says that a larger value of v provides at least as much evidence of greater effort than a smaller value of v does. In notation, this condition writes as

$$\frac{\partial}{\partial v}\left[\frac{f_e(v; r, e)}{f(v; r, e)}\right] \geq 0. \tag{S1.5}$$

Crucially, the MLRP for effort extends to $v = 0$. The fact that the MLRP for effort extends to $v = 0$ while the MLRP for risk does not creates an unavoidable tension between the incentives for risk-taking and the incentives for effort that stymies the community's ability to motivate both efficiently.

S1.2. Full information

Under full information, the community's problem is only constrained by the monotonicity constraint MC and budget constraint BC. To show this formally, assume that the MC will hold. Using $\beta \geq 0$ as the multiplier on the BC, the Lagrangian for sub-problem S1.1 writes as

$$L(\bar{u}^{(r,e)}(\cdot), \beta; r, e) = \int \bar{u}^{(r,e)}\, F(dv; r, e) - c(e) + \beta \left[\bar{B}(r, e) - \int h(\bar{u}^{(r,e)})\, F(dv; r, e) \right].$$

with the slackness condition $\beta \left[\bar{B}(r,e) - \int h(\bar{u}^{(r,e)}(v))\, F(dv; r, e) \right] = 0$. Set $\partial L / \partial \bar{u}^{(r,e)}(v) = 0$ and differentiate pointwise to give

$$0 = f(v; r, e) \left[1 - \beta h'(\bar{u}^{(r,e)}(v)) \right].$$

Thus $h'' > 0$ implies $\bar{u}^{(r,e)}(v) = \bar{u}^{(r,e)}$ is constant for all v, and $h' > 0$ implies $\beta = 1/h'(\bar{u}^{(r,e)}) > 0$, thus the BC binds. Note that β (the shadow price of the budget constraint) equals $u'(w)$, the marginal utility of wages at equilibrium; a similar interpretation of β will hold when the action is hidden. If $\bar{u}^{(r,e)}(v)$ is constant and the BC binds, then every investigator receives an equal wage $w = \bar{B}(r,e)$. Thus the community contracts on the action that maximizes $u(\bar{B}(r,e)) - c(e)$.

In general, it is not necessarily the case that the community will contract on the progress-maximizing action. However, because risk-taking is not intrinsically onerous, the community will contract on the progress-maximizing level of scientific risk for any particular effort level. That is, for any e, $\arg\max_r u(\bar{B}(r,e)) = \arg\max_r s(r,e)$. This follows from $u' > 0$ and $B' > 0$.

S1.3. Analysis of the general model

We first consider the general version of the model in which effort may affect both the probability of obtaining a publishable outcome and the distribution of v for publishable outcomes. In this model, there are several binding constraints, so we must check that the constraint qualification holds. It is straightforward to show that the only way in which the qualification would not hold is if $f_e(v; r, e)/f_r(v; r, e)$ is constant for all v.[1]. We assume that this will not be the case; it is easy to verify as much for the numerical example.

Use λ and μ as the multipliers for EC and RC, respectively, and assume that the MC will hold. The Lagrangian for sub-problem S1.1 writes as

$$\begin{aligned} L(\bar{u}^{(r,e)}(\cdot), \beta, \lambda, \mu; r, e) = &\int \bar{u}^{(r,e)}(v)\, F(dv; r, e) - c(e) + \beta \left[\bar{B}(r,e) - \int h(\bar{u}^{(r,e)}(v))\, F(dv; r, e) \right] \\ &+ \lambda \left[\int \bar{u}^{(r,e)}(v)\, F_e(dv; r, e) - c'(e) \right] + \mu \int \bar{u}^{(r,e)}(v)\, F_r(dv; r, e) \end{aligned} \tag{S1.6}$$

with $\beta > 0$ and the slackness condition $\beta \left[\bar{B}(r,e) - \int h(\bar{u}^{(r,e)}(v))\, F(dv; r, e) \right] = 0$. We have reversed the sign of λ and μ from the way they are typically written to ease the forthcoming interpretation. As before, set $\partial L / \partial \bar{u}^{(r,e)}(v) = 0$ to give

$$0 = f(v; r, e) - \beta h'(\bar{u}^{(r,e)}(v)) f(v; r, e) + \lambda f_e(v; r, e) + \mu f_r(v; r, e). \tag{S1.7}$$

Integrate with respect to v obtain

$$0 = \int F(dv; r, e) - \beta \int h'(\bar{u}^{(r,e)}(v)) F(dv; r, e) + \lambda \int F_e(dv; r, e) + \mu \int F_r(dv; r, e).$$

[1] The constraint qualification holds if the constraints are linearly independent. Assuming the BC will bind, the qualification is violated if we can find a contract $\bar{u}(v)$ and values η_1, η_2, and η_3, not all 0, such that $\eta_1 h'(\bar{u}(v)) f(v; r, e) + \eta_2 f_e(v; r, e) + \eta_3 f_r(v; r, e) = 0$ for all $v \geq 0$. Integrating over $v \geq 0$ gives $\eta_1 \int h'(\bar{u}(v)) F(dv; r, e) + \eta_2 \int F_e(dv; r, e) + \eta_3 \int F_r(dv; r, e) = 0$, from which $\int F_e(dv; r, e) = \int F_r(dv; r, e) = 0$ implies $\eta_1 = 0$. Thus the constraint qualification is only violated if $f_e(v; r, e)/f_r(v; r, e)$ is constant for all v.

Now use $\int F(dv;r,e) = 1$ and $\int F_e(dv;r,e) = \int F_r(dv;r,e) = 0$ to obtain

$$\beta^{-1} = \int h'(\bar{u}^{(r,e)}(v))F(dv;r,e)$$

from which $\beta > 0$ and the BC binds, as before.

Now divide eq. S1.7 by $f(v;r,e)$ and rearrange to yield

$$\beta h'(\bar{u}^{(r,e)}(v)) - 1 = \lambda \frac{f_e(v;r,e)}{f(v;r,e)} + \mu \frac{f_r(v;r,e)}{f(v;r,e)}. \tag{S1.8}$$

By $\beta > 0$, the MC, and $h'' > 0$, the LHS of eq. S1.8 must be non-decreasing in v. We can rule out $\lambda = \mu = 0$, because this would give a constant wage for all v, and a constant wage cannot implement costly effort. By our assumptions about the MLRP, $f_e(v;r,e)/f(v;r,e)$ is non-decreasing in v for all v, while $f_r(v;r,e)/f(v;r,e)$ is not monotone (it is decreasing in v at $v = 0$, and strictly increasing in v for $v > 0$). Thus we must have $\lambda > 0$,[2] and the EC binds.

Without further assumptions on the structure of $F(v;r,e)$, we cannot take the analysis any further. Most notably, the sign of μ is ambiguous, and we will see that establishing $\mu > 0$ is key to the proof below that the community distorts risk downwards at equilibrium in the special case of eq. 4.

S1.4. Analysis of the special case

Here, we show that with the additional assumption of eq. 4 the community's tradition is guaranteed to distort risk-taking downwards from its productivity-maximizing level. The basic path of the proof is to show that when effort does not affect the conditional distribution of v for publishable outcomes, $f_e(v;r,e)/f(v;r,e)$ is constant for $v > 0$, which in turn implies $\mu > 0$ by eq. S1.8. By applying the Envelope Theorem it can be shown that $\mu > 0$ implies $s_r(\tilde{r},\tilde{e}) > 0$. Because we have assumed that $s(r,e)$ is strictly concave in r for any e, it then follows that $\tilde{r} < \arg\max_r s(\tilde{e},r)$. Finally, because $s(r,e)$ is separable in e and r under eq. 4, it follows that $\arg\max_r s(r,e) = \hat{r}$ for any e, and thus $\tilde{r} < \hat{r}$.

Let $f(v;r) = f(v;e=1,r)$ give the density of v under full effort ($e = 1$). Under eq. 4, the full density of v can be written

$$f(v;r,e) = \begin{cases} 1 - e(1 - f(0;r)) & v = 0 \\ ef(v;r) & v > 0 \end{cases}$$

Thus, the likelihood ratio with respect to e is constant for $v > 0$, that is, $f_e(v;r,e)/f(v;r,e) = 1/e$. By eq. S1.8, it then follows that $\mu > 0$ and the RC binds.[3]

Write the investigators' payoff at the optimal contract for implementing action (r,e) as $\Pi(r,e) = \pi(r,e,\bar{u}^{(r,e)}(\cdot))$. Apply the Envelope Theorem [33, p. 456] to give

$$\begin{aligned}\frac{\partial \Pi(r,e)}{\partial r} &= \int \bar{u}^{(r,e)}(v)\, F_r(dv;r,e) + \beta \left[\bar{B}_r(r,e) - \int h(\bar{u}^{(r,e)}(v))\, F_r(dv;r,e)\right] \\ &\quad + \lambda \left[\int \bar{u}^{(r,e)}(v)\, F_{re}(dv;r,e)\right] + \mu \int \bar{u}^{(r,e)}(v)\, F_{rr}(dv;r,e).\end{aligned} \tag{S1.9}$$

The first term on the RHS in S1.9 vanishes because $\bar{u}^{(r,e)}(v)$ implements r, and hence $\frac{\partial}{\partial r} \int \bar{u}^{(r,e)}(v)\, F(dv;r,e) = 0$ by

[2] Suppose not. If $\lambda = 0$, there is no choice of $\mu \neq 0$ that would make the RHS of eq. S1.8 non-decreasing in v, because $f_r(v;r,e)/f(v;r,e)$ is not monotone. If $\lambda < 0$, we would need $\mu < 0$ to make the RHS of eq. S1.8 non-decreasing at $v = 0$, yet $\lambda < 0$ and $\mu < 0$ would make the RHS of eq. S1.8 strictly decreasing in $v = 0$ for $v > 0$. Note also that λ is the shadow price of the EC; thus $\lambda > 0$ indicates that (reasonably) the payoff decreases as the EC tightens.

[3] We have already argued that the LHS of eq. S1.8 must be non-decreasing in v for all $v \geq 0$. Further, $\bar{u}^{(r,e)}(v)$ must be strictly increasing in v for at least some $v > 0$; otherwise, the contract could only implement the minimal scientific risk $r = 0$. Thus the LHS of eq. S1.8 must be strictly increasing in v for some $v > 0$ as well. But if $f_e(v;r,e)/f(v;r,e)$ is constant for $v > 0$, this requires $\mu > 0$.

the RC. Under eq. 4, the term in square brackets that multiplies λ in eq. S1.9 also vanishes:

$$\begin{aligned}\int \bar{u}^{(r,e)}(v)\, F_{re}(dv;r,e) &= \frac{\partial^2}{\partial e\,\partial r}\int \bar{u}^{(r,e)}(v)\, F(dv;r,e)\\ &= \frac{\partial^2}{\partial e\,\partial r}\left[\bar{u}^{(r,e)}(0)(1-e) + e\int \bar{u}^{(r,e)}(v)\, F(dv;r)\right]\\ &= \frac{\partial}{\partial r}\int \bar{u}^{(r,e)}(v)\, F(dv;r)\\ &= 0 \end{aligned} \tag{S1.10}$$

where the last equality follows from the RC.[4] In other words, the mixed partial $\partial^2\Pi(r,e)/\partial e\partial r = 0$. The implication is that, for any contract that implements a particular action, a marginal change in risk-taking away from the implemented action has no effect on the sensitivity of the payoff to effort, or vice versa.

Continuing with the proof, set $\partial\Pi(\tilde{r},\tilde{e})/\partial r = 0$ and re-arrange the remaining terms in S1.9 to give

$$\beta \bar{B}_r(\tilde{r},\tilde{e}) = \beta\int h(\bar{u}^{(\tilde{r},\tilde{e})}(v))\, F_r(dv;\tilde{r},\tilde{e}) - \mu\int \bar{u}^{(\tilde{r},\tilde{e})}(v)\, F_{rr}(dv;\tilde{r},\tilde{e}). \tag{S1.11}$$

On the left of eq. S1.11, note that $\bar{B}_r(\tilde{r},\tilde{e}) = dB(s(\tilde{r},\tilde{e}))/dr = B'(s(\tilde{r},\tilde{e}))s_r(\tilde{r},\tilde{e})$. On the right, $\int \bar{u}^{(\tilde{r},\tilde{e})}(v)\, F_{rr}(dv;\tilde{r},\tilde{e}) = \frac{\partial^2}{\partial r^2}\int \bar{u}^{(\tilde{r},\tilde{e})}(v)\, F(dv;\tilde{r},\tilde{e}) \le 0$, because $\bar{u}^{(\tilde{r},\tilde{e})}(v)$ maximizes the community's payoff when implementing $(\tilde{r},\tilde{e})$. Thus it suffices to show that $\int h(\bar{u}^{(\tilde{r},\tilde{e})}(v))\, F_r(dv;\tilde{r},\tilde{e}) > 0$, because this together with $B' > 0$, $\beta > 0$, and $\mu > 0$ implies that $s_r(\tilde{r},\tilde{e}) > 0$, which in turn implies that risk-taking is distorted downwards at equilibrium.. The following lemma provides the needed result.

Lemma: For any action, $\frac{d}{dr}\int h(\bar{u}^{(r,e)}(v))\, F(dv;r,e) > 0$.

Proof. The claim of the lemma is that if investigators increase their scientific risk marginally when faced with a contract that implements (r,e), then the total wage payouts will increase also. The intuition is that because $\bar{u}^{(r,e)}(\cdot)$ implements (r,e), then by the RC a marginal increase in risk does not change the investigators' wage utility, that is, $\frac{d}{dr}\int \bar{u}^{(r,e)}(v)\, F(dv;r,e) = 0$. However, the wages that need to be paid to create this wage-utility schedule are a convex in the wage utility, that is, $h'' > 0$. Thus, because increasing risk increases the variance in the scientific value of the outcome, a marginal increase in risk increases the total wages paid. The rest is just working out the mathematical details.

To ease the notation in the proof, suppress the (r,e) superscript on $\bar{u}(v)$. By MLRP, there is a value $v_0 > 0$ such that[5]

$$f_r(v;r,e)\begin{cases} < 0 & 0 < v < v_0\\ = 0 & v_0\\ > 0 & v_0 < v.\end{cases}$$

By RC, we have

$$\begin{aligned} 0 &= \int_{v\ge 0}\left[\bar{u}(v) - \bar{u}(0) + \bar{u}(0)\right]\, F_r(dv;r,e)\\ &= \int_{v\ge 0}\left[\bar{u}(v) - \bar{u}(0)\right]\, F_r(dv;r,e) + \bar{u}(0)\int_{v\ge 0} F_r(dv;r,e)\\ &= \int_{v>0}\left[\bar{u}(v) - \bar{u}(0)\right]\, F_r(dv;r,e)\\ &= \int_{v\in(0,v_0)}\left[\bar{u}(v) - \bar{u}(0)\right]\, F_r(dv;r,e) + \int_{v>v_0}\left[\bar{u}(v) - \bar{u}(0)\right]\, F_r(dv;r,e)\end{aligned}$$

---

[4] To see this, RC implies $0 = \frac{\partial}{\partial r}\int \bar{u}^{(r,e)}(v)\, F(dv;r,e) = \frac{\partial}{\partial r}\left[\bar{u}^{(r,e)}(0)(1-e) + e\int \bar{u}^{(r,e)}(v)\, F(dv;r)\right] = e\frac{\partial}{\partial r}\int \bar{u}^{(r,e)}(v)\, F(dv;r)$, and thus $\frac{\partial}{\partial r}\int \bar{u}^{(r,e)}(v)\, F(dv;r) = 0$ as long as $e > 0$.

[5] Actually, the MLRP does not rule out the possibility that $f_r(v;r,e) < 0$ for all $v > 0$. But if this were the case, then RC would require that $\bar{u}(v) = \bar{u}(0)$ for all $v \ge 0$, which cannot implement costly effort. So we know that $f_r(v;r,e) > 0$ for some $v > 0$.

Now, because we are only integrating over values $v > 0$, write $F_r(dv; r, e) = f_r(v; r, e)\, dv$ and interpret the integrals as Riemann integrals. Divide through by $\bar{u}(v_0) - \bar{u}(0) > 0$ to give

$$0 = \int_{v\in(0,v_0)} \frac{\bar{u}(v) - \bar{u}(0)}{\bar{u}(v_0) - \bar{u}(0)}\, f_r(v; r, e)\, dv + \int_{v>v_0} \frac{\bar{u}(v) - \bar{u}(0)}{\bar{u}(v_0) - \bar{u}(0)}\, f_r(v; r, e)\, dv.$$

Now by the convexity of h, we have

$$\frac{h(\bar{u}(v)) - h(\bar{u}(0))}{h(\bar{u}(v_0)) - h(\bar{u}(0))} < \frac{\bar{u}(v) - \bar{u}(0)}{\bar{u}(v_0) - \bar{u}(0)} \text{ for } v < v_0$$

and

$$\frac{h(\bar{u}(v)) - h(\bar{u}(0))}{h(\bar{u}(v_0)) - h(\bar{u}(0))} > \frac{\bar{u}(v) - \bar{u}(0)}{\bar{u}(v_0) - \bar{u}(0)} \text{ for } v > v_0.$$

Thus

$$\int_{v\in(0,v_0)} \frac{\bar{u}(v) - \bar{u}(0)}{\bar{u}(v_0) - \bar{u}(0)}\, f_r(v; r, e)\, dv < \int_{v\in(0,v_0)} \frac{h(\bar{u}(v)) - h(\bar{u}(0))}{h(\bar{u}(v_0)) - h(\bar{u}(0))}\, f_r(v; r, e)\, dv < 0$$

and

$$0 < \int_{v>v_0} \frac{\bar{u}(v) - \bar{u}(0)}{\bar{u}(v_0) - \bar{u}(0)}\, f_r(v; r, e)\, dv < \int_{v>v_0} \frac{h(\bar{u}(v)) - h(\bar{u}(0))}{h(\bar{u}(v_0)) - h(\bar{u}(0))}\, f_r(v; r, e)\, dv$$

from which

$$\begin{aligned} 0 &< \int_{v\in(0,v_0)} \frac{h(\bar{u}(v)) - h(\bar{u}(0))}{h(\bar{u}(v_0)) - h(\bar{u}(0))}\, f_r(v; r, e)\, dv + \int_{v>v_0} \frac{h(\bar{u}(v)) - h(\bar{u}(0))}{h(\bar{u}(v_0)) - h(\bar{u}(0))}\, f_r(v; r, e)\, dv \\ &= \frac{1}{h(\bar{u}(v_0)) - h(\bar{u}(0))} \int [h(\bar{u}(v)) - h(\bar{u}(0))]\, F_r(dv; r, e) \\ &= \frac{1}{h(\bar{u}(v_0)) - h(\bar{u}(0))} \int h(\bar{u}(v))\, F_r(dv; r, e) \end{aligned}$$

and the result follows. □

S1.5. Numerical example

In the numerical examples of Figs. 2 and 3, the quantities $f_e(v; r, e)/f(v; r, e)$ and $f_r(v; r, e)/f(v; r, e)$ are both linear in v for $v > 0$. Specifically, we have $f_e(v; r, e)/f(v; r, e) = v/(2re^{3/2})$ while $f_r(v; r, e)/f(v; r, e) = v/(r^2\sqrt{e}) - 1/r - 1/(1 - r)$ for $v > 0$. Consequently, using S1.8 along with $h'(u) = 2u$ establishes that $\bar{u}^{(r,e)}(v)$ is linear in v for $v > 0$, and thus can be written

$$\bar{u}^{(r,e)}(v) = \begin{cases} \bar{u}_0 & v = 0 \\ a + bv & v > 0. \end{cases}$$

Thus $\bar{u}^{(r,e)}(v)$ is fully determined by the triple $(\bar{u}_0, a, b)$. The (binding) BC, EC, and RC thus create a system of three equations in three unknowns that can be solved numerically to find the contract $\bar{u}^{(r,e)}(v)$, which solves the first sub-problem. To solve the second sub-problem, the optimal values of e and r are then found by using the "optimize" routine in the R computing environment [34]. The "optimize" routine is based on the Algol 60 procedure given in ref. [35]. The second-order conditions for the second sub-problem are verified numerically at the computed optimum.
